# Does corporate governance differ by sector? An analysis under good practice criteria. The case of Spain

**DOI:** 10.1371/journal.pone.0307806

**Published:** 2024-10-11

**Authors:** Javier Corral-Lage, Asier Garayeta, Eduardo Trigo, J. Iñaki De la Peña

**Affiliations:** 1 Financial Economics I Department, Faculty of Business Administration, University of the Basque Country (UPV/EHU), Bilbao, Spain; 2 Finance and Accounting Department, University of Malaga (UMa), Málaga, Spain; University of Almeria: Universidad de Almeria, SPAIN

## Abstract

The aim of this paper is to analyse how the factors remuneration, supervision and board structure influence the good corporate governance of companies in the Spanish Continuous Market. This paper develops, for the first time, an index based on the recommendations defined in the Good Governance Code of Listed Companies. This paper measures remuneration, monitoring and governance structure and employs a multiple linear regression model modelling corporate governance as a latent factor. Based on this model, this research presents empirical evidence of the relationship between corporate governance and the defined variables, considering Spanish Continuous Market firms disaggregated by sector. To date, there are no studies that have taken into account the analysis for all sectors in the same country. Among the conclusions, the research finds that the larger a company is, the better the mechanisms for providing an optimal degree of governance, as is the case in the oil, energy and technology sectors. In another sense, the higher the number of proprietary directors the lower the levels of good governance, mainly in basic materials, industrial, construction and consumer goods and services companies. The empirical results also justify the inclusion of Corporate Governance-related factors in governance regulation.

## 1 Introduction

Corporate governance (CG) is a combination of policies, laws and instruments involved in the management and governance of a company [1]. It is a set of rules that ensures a relationship between a company and its stakeholders is transparent and equitable [2, 3]. In this way, good CG makes companies attractive to future investors and increases market confidence [4].

Corporate scandals and the 2008 financial crisis raised awareness that corporate governance is a significant issue for all institutions, but especially for companies. The lack of oversight by boards of directors and the lack of impact of supervision on corporate governance were detrimental to the reliability of companies and financial markets. Since the financial crisis, companies are more consistent with the need for sound governance structures and good governance systems [5].

The predominant theoretical framework in CG studies [6–9] is agency theory [10]. Its popularity is due to two characteristics: a) it reduces decision making to two groups—management and shareholders—with clearly defined divergent interests [11]; and b) assumes that human beings are intrinsically selfish [12] and therefore every rational individual pursues their own interests.

However, there are other theories related to good CG. Management Theory states that there are ethical and professional motives that will prevent conflicts of interest from developing between the two parties [13]. The Resource Dependence Theory [14, 15] argues that a board well connected to its external environment will have easier access to resources, such as financial resources, which could then be allocated to sustainable actions. The Theoretical Institutional Perspective [16] if governance aims for legitimacy over economic efficiency [17], then social welfare and stakeholder balance will be central [18, 19]. Finally, based on stakeholder’s theory [20, 21] the Board of Directors should always take into account all stakeholders who may have any kind of interest in the company.

Corporate agency conflicts have been the subject of extensive research in recent decades, much of which has focused on the alignment of interests between shareholders and corporate management [22]. The main mechanisms for achieving alignment are to be found in the field of CG, the subject of the research presented in this paper. Using factors identified as potentially mitigating agency conflicts [23], this paper investigates the influence of compensation, monitoring and board structure [22] in relation to Spanish Continuous Market (SCM) firms.

These conflicts are related to the good governance of an organisation and its efficient functioning [22]. Mechanisms employed in good CG constrain managers’ self-interests to be aligned with those of shareholders [24]. In most companies, this internal mechanism is a well-structured board of directors [25] and a pay structure that orients the manager towards shareholder interests [26]. If the internal mechanisms of good CG are properly implemented, it reduces investor risk, thereby increasing investment capital and improving both financial and sustainable business performance [27].

This has been widely debated, using different measures to analyse its effect on firm performance, which can be operational, market or financial [28]. When analysing the components that influence CG, most studies have focused on analysing the variables that affect CG through private indices [29–36].

This paper, as a novelty, analyses which variables referenced in the literature do or do not influence good CG through the elaboration, for the first time, of a public index based on the Good Governance Code of Listed Companies (CBGC) [37]. This report indicates the degree of compliance with the recommendations given by the European Parliament and the European Council on good corporate governance [38]. This will be referred to as the Corporate Governance Rating Index (CGRI), developed in the methodology section.

To date, several studies have investigated the influence of different variables on governance, but focusing exclusively on one sector, e.g. the tourism sector [39]; the health sector [40]; the financial sector [41]; the insurance sector [22], etc. This paper provides an in-depth analysis of the relationships between the factors remuneration, oversight and board structure, disaggregating and analysing the data according to the sector in which the SCM companies are grouped. Thus, the present research analyses seven sectors, the first time such an in-depth analysis of a country has been carried out.

There are interrelationships between sustainability and CG that have been reflected, among others, in corporate social responsibility and reporting, corporate governance strategies, and board composition [42]. In the specific case of remuneration, transparency issues can lead to sustainability problems as most remuneration policies tend to be oriented towards financial targets [43]. On the other hand, monitoring has increased in the last decade, and has even been incorporated into social and environmental measurement. Its purpose is to find corporate sustainability [42]. The same symbiosis is taking place between corporate governance structure and its financial and sustainability performance by supporting broader stakeholder participation [44].

The aim of this paper is to analyse how the factors compensation, monitoring and board structure influence the good corporate governance of SCM companies in relation to the CGRI. This research uses a multiple linear regression model to measure the relationship between good corporate governance (CG) and the defined variables, considering for the first time in the literature, the SCM companies disaggregated by sector of activity.

To address this objective, the paper is structured as follows. The second section reviews the measurement of CG. It locates the establishment of an index based on public information and the key factors that the literature considers relevant for good CG. Next, the research data and methodology of the present research is presented, determining for the Spanish case the variables that can help to measure the corporate governance of SCM companies, and the empirical model followed by a discussion of the sectoral results. The fourth explains the results obtained through the empirical research, both at a global level and by productive sector; and ends with the discussion and most relevant conclusions based on the data and references used.

## 2 Literature and hypothesis development

### 2.1 Index for measuring corporate governance

Good governance is based on transparency [2, 3], providing useful information to shareholders [45] and confidence about the companies in which they invest [46, 47]. It also facilitates investment in companies [48] and enhances corporate reputation [49] by giving it legitimacy in the eyes of stakeholders and society [50].

Furthermore, good governance processes are based on accountability [1, 51] and long-term focus [52]. This enables higher performance [53, 54] and helps foster growth [55] and business stability [56].

Optimal and transparent CG is essential not only to increase competitiveness [57–59] and business efficiency, but also to strengthen the protection of shareholders’ and third parties’ rights.

The European Parliament (2012) [38] posits that optimal CG is first and foremost the responsibility of the company [60] and there are standards at national and European Union (EU) level to ensure that certain criteria are met at the level of good CG. In addition, various ratings can be used to assess an entity’s corporate governance (Table 1).

**Table 1:** Private ratings for assessing corporate governance.

Authors	Index	Explanation
**Khanchel (2007) [34]**	Standard & Poor’s	List of 80–100 factors. It is grouped into three categories: ownership structure and investor relations, financial transparency and disclosure, and board and management structure and process.
**Bauer et al. (2004) [30]**	Eurotop 300 del Financial Times Stock Exchange (FTSE)	It is based on some 300 different criteria. These criteria can be grouped into four broader categories: shareholder rights and duties, range of takeover defences, CG disclosure and board structure and functioning.
**Klapper & Love (2004) [31]**	Credit Lyonnais Securities Asia-CLSA Ltd.	The ranking takes into account seven categories: discipline, transparency, independence, accountability, responsibility, impartiality and social conscience. Each category has a weight of 0.15, except for the last one, which has a weight of 0.10.
**Brown & Caylor (2006) [33]**	Shareholder Services	The elements are divided into four equally weighted categories (0.25): shareholder rights, board of directors, external directors, and disclosure and transparency.

In recent years there has been a proliferation of initiatives related to good corporate governance practices, the intensity of which has multiplied since the onset of the international financial crisis, due to the widespread conviction of the importance of listed companies being properly and transparently managed as an essential factor for generating value in companies, improving economic efficiency and reinforcing investor confidence [37].

Many EU countries have recently adopted codes of good practice to establish guidelines for listed companies to improve the overall quality of CG. Spain has been no stranger to this movement, with notable progress having been made in the area of good corporate governance. In Spain, the government offers recommendations subject to the principle known internationally as "comply or explain" to classify the specific CG of each company in the corporate governance framework [61]. This means that companies that do not comply with part of the CGRI requirements must explain why. While full compliance can send a positive signal to the market [3] and to society [62], it may not always be the best approach for a company from a CG perspective [63]. In certain cases, not implementing a provision allows for more effective management of the company [64, 65].

The use of good governance codes of a public nature together with the "comply or explain" principle are a useful system to achieve part of the objectives of good CG and therefore is the system consistently followed both in the main countries of the European Union and in other developed countries [7, 66, 67]. It thus highlights its flexibility in the way it is applied and the possibility of constituting a reference of good corporate governance practices. Furthermore, the European Union expressly includes in its regulations the validity of this principle of action, recently confirmed in the EU Green Paper on corporate governance of listed companies [65].

Studies that have included CG index [7, 66, 67] have designed their index considering CG facets in isolation. One of the contributions of this paper is to consider these facets together. Furthermore, this research designs a specific index based on data that are public and mandatory. This information has to be compulsorily reported by the SCM companies, for the measurement of CG.

Thus, the CGRI used in this research is based on monitoring the recommendations of public institutions [68, 69]. The 64 recommendations defined are grouped into three main blocks:

- General aspects (statutory limitations, company listing, monitoring of recommendations, shareholders’ and block shareholders’ meetings, share issues).
- General meeting of shareholders (transparency, attendance and participation, attendance fees policy).
- Board (responsibility, structure and composition, functioning and organisation of the board; remuneration of directors; sustainability, environmental and social aspects).

Once the criteria are known, companies only have to report information on whether they meet these criteria, either affirmatively, negatively, partially or not at all, or whether the criteria do not apply [31, 33]. The answer reported in this way is categorical and thus quantified, so that it can be processed and its result can be incorporated in the creation of the index.

The analysis developed in this paper is important in light of recent financial scandals and crises, which have highlighted the link between corporate governance and the remuneration systems, supervision and structure of corporate boards.

The underlying theory of corporate governance is that systems can be put in place to align the incentives of investors and management. These systems typically include the methods of remuneration of board members (often referred to as internal incentives), the mechanisms available to shareholders to control the behaviour of board members, and the ownership structure [23].

Therefore, in our model, we analyse the influence of compensation (board remuneration), oversight (number of independent directors, number of board meetings and number of female directors) and ownership structure (number of block shareholders and number of proprietary members) on the basis of CG.

All three elements are important in reducing agency conflicts between investors and executives, which arise due to the separation between decision-making and control of decisions [22, 23, 70, 71]. In addition, all three factors have been identified in the literature as relevant for the achievement of optimal governance, for that reason will determine the hypothesis.

### 2.2 Compensation

Higher wages are associated with less risky decisions and thus with better CG [72]. Interests between investors and executives can be aligned through compensation systems. An important aspect in this context is the level of executive remuneration compared to the market average. In a free market with utility-maximising managers, managers work for firms where they receive the highest utility. In the light of utility theory, the level of pay could be positively correlated with corporate governance [73, 74].

The remuneration of directors and senior management can go against other stakeholders’ interests [75]. It is therefore recommended that their remuneration be monitored and controlled [76]. In the case of directors, this is achieved by limiting the remuneration amount and by restricting share distribution [77]. In the case of senior managers, the greater the corporate control, the lower the managerial remuneration and the less it is linked to results [78]. Furthermore, executive cash compensation is negatively associated with CG, so this type of remuneration is not recommended [72]. Furthermore, in some countries, such as Finland, it is shown that there are links between remuneration and sustainability [43].

Based on the analysis presented above, the following hypothesis should be analysed:

**H1**: *There is no relationship between adequate GC and compensation (COM)*.

### 2.3 Monitoring

Supervision by the board of directors is seen as an important corporate governance mechanism and a means for shareholder influence [79]. The composition of boards in companies according to gender, age or profession are determinant in the organisation and sustainability performance [44].

Multiple research has focused on the independence of boards for adequate CG oversight [80]. In many cases, a positive relationship has also been observed between more rigorous oversight of a company’s management and governance with a board with mostly independent members [80] and more efficient meetings [81]. In addition, the number of female board members influences oversight [82] and their inclusion improves governance [83].

Based on the analysis presented above, the following hypothesis should be analysed:

**H2**: *There is no positive relationship between adequate CG and its monitoring*.

### 2.4 Structure of boards

Previous studies have shown that there is a positive relationship between blockholders—shareholders who own more than 5% of the share capital—and governance in relation to promoting efficiency [84]. However, studies suggest that control of a company’s capital in the hands of a few can lead to confrontation with management and hinder good governance [22]. These tensions can emerge between the shareholder and the board when they assume postulates to achieve greater CG oriented towards sustainability [42].

Some authors accuse proprietary directors of focusing exclusively on enriching themselves by owning a significant stake in the entity’s capital without worrying about the development of good governance in the company [85, 86]. The task of the board director is not only to maximise the well-being of shareholders but also to seek an ethical approach to stakeholders [42].

So, based on the analysis presented above, the following hypothesis should be analysed:

**H3**: *There is no positive relationship between adequate CG and governance structure*.

## 3 Data and method

### 3.1 Data

In Spain, the obligation to report non-financial information was established in 2017 and came into force in 2018 [69]. The CGRI thus contains an analysis of the importance of good CG practices to increase economic efficiency and strengthen investor confidence. It provides an overview of the evolution of CG rules at EU and international level, a summary of the main regulatory developments and includes recommendations of the codes of good governance as well as a description of the listed companies’ CG guidelines.

The database therefore starts with the SCM for the years 2018 and 2019. The years 2020 and 2021 have been discarded to avoid the effect of the COVID-19 pandemic on the variables considered. As of the date of the present research, not all companies surveyed have published their 2022 data. Four companies have been eliminated because they were either not subject to Spanish legislation or their data contained biases or errors. The data sources used for the sample are from the Annual Corporate Governance Reports (ACGR) and the CNMV’s reports, which are also published annually.

The CGRI (Table 2) was obtained using the 64 items over two years for 101 listed companies on the SCM. Thus, 12,928 observations were used. In addition, through the accounting information, the sample has a panel of 2020 data, corresponding to 1010 annual data (101 companies x 10 variables/independent companies), standardised in order to eliminate the period impact.

**Table 2:** Descriptive statistics of the CGRI.

	N	Minimum	Maximum	Media	Deviation
CGRI	202	,66796875	,98046875	,8823217651	,07065246626

To enable the present research to be carried out, the analysed companies’ descriptive data were broken down according to sector, in accordance with the CNMV (Table 3):

**Table 3:** Frequencies of the variable SECTOR.

Sector	Frequency	Percentage	Cumulative percentage
1	16	7,8	7,9
2	53	26,0	34,2
3	45	22,1	56,4
4	28	13,7	70,3
5	24	11,8	82,2
6	12	5,9	88,1
7	24	11,8	100,0
**Total**	**202**	**100,0%**	

(1) Oil and energy; (2) Basic materials, industry and construction; (3) Consumer goods; (4) Consumer services; (5) Financial services; (6) Technology and telecommunications; (7) Real estate services.

Table 3 shows that the main sector in the SCM is basic materials, industry and construction, followed closely by consumer goods. Multiple linear regression is used to test the indicated hypotheses. In this way the interaction of the variables is analysed, which determines the relationship of the three defined blocks as far as CG is concerned.

The CG measurement is based on the 64 items established for the CGRI for each of the companies analysed (101) for each year. Thus, each recommendation has a specific weighting of:

a) 1 if the recommendation is explained or implemented;
b) 0.75 if not implemented;
c) 0.5 if partially implemented; in case of non-implementation or no concrete and concise explanation of the recommendation;
d) 0 if the recommendation is not compliant

The quantification of the 64 criteria is based on the good practice guide for the application of the comply-or-explain principle that was adopted on 15 July 2019. In this way, the 4 categories of each of the 64 criteria are evaluated per company. A value is thus constructed that allows different companies to be compared on the basis of the information provided.

### 3.2 Incorporation of variables into the model

The most relevant CG measurement factors are remuneration, monitoring and governance structure. However, instead of focusing on only three variables, the model has been enhanced to incorporate a larger number of variables, by calculating the dependent and explanatory variables in the following way.

#### Executive compensation or remuneration (COM) (V_1_) [73, 74]

To determine compensation or remuneration, executive compensation is taken into account: Remuneration for membership of the Board and/or Board Committees, Salaries Variable cash remuneration, Share-based remuneration systems, Bonuses and Long-term savings schemes published in each of the financial reports of the companies analysed by year. Thus, more than a thousand observations have been used to derive this factor. The full amount paid by the company to its executives is taken into account, regardless of how many directors there are (compensation per executive). Likewise, for board compensation, calculations are made to obtain the total compensation per board member on a logarithmic scale basis.

#### Monitoring

Monitoring of the board of directors is fundamental to corporate governance, as the board is the chief decision-making body of the company. Effective board monitoring can help ensure that decisions are made in a fair and equitable manner, and that the interests of all shareholders are protected. Torchia et al. (2015) [87] determined the relationship between board monitoring and the quality of corporate governance in Italian companies. The authors found that board oversight was positively related to corporate governance quality.

Board monitoring is a factor that is often measured in the literature through the use of several variables:

- Number of council meetings throughout the year [88–91] ***(NRC) (V***_***2***_***)***.
- Ratio of independent board members: percentage independent board members to total board members [22] ***(IND) (V***_***3***_***)***. Research has shown that increasing the number of board members can work against sustainability [92].
- Ratio of executive board members [84, 93, 94]: percentage of executives belonging to a company who are part of the top management ***(EJ) (V***_***4***_***)*.**
- Ratio of female directors: percentage women to total board members ***(NCA) (V***_***5***_***)*** [79].

#### Governance structure

Management involvement in ownership aligns management and shareholders’ interests [95, 96]. Moreover, they can legitimise their decisions through senior management. There are also situations where company ownership is highly concentrated and management will be under the scrutiny of shareholders, which limits the decision-making power as well as the policies and strategies to be pursued.

Previous studies have shown that there is a positive relationship between blockholders ***(ADV) (V***_***6***_***)*** and CG in relation to promoting efficiency [84]. However, it is true that an increase in the number of blockholders increases the number of investors and that a company’s portfolio is not diversified [22]. On the other hand, some authors [85, 86] accuse blockholders of extracting profits for priority shareholders.

The variables related to blockholders allow the number of significant company shareholders to be analysed [97]. In Spain, those who hold 5% or more of a company’s shares can influence company decision-making ***(DOM) (V***_***7***_***)***. The analysis is therefore carried out by studying the percentages of shareholders with voting rights and the percentage of proprietary board members, as the latter can have an outside influence on a company.

**Fig 1:**
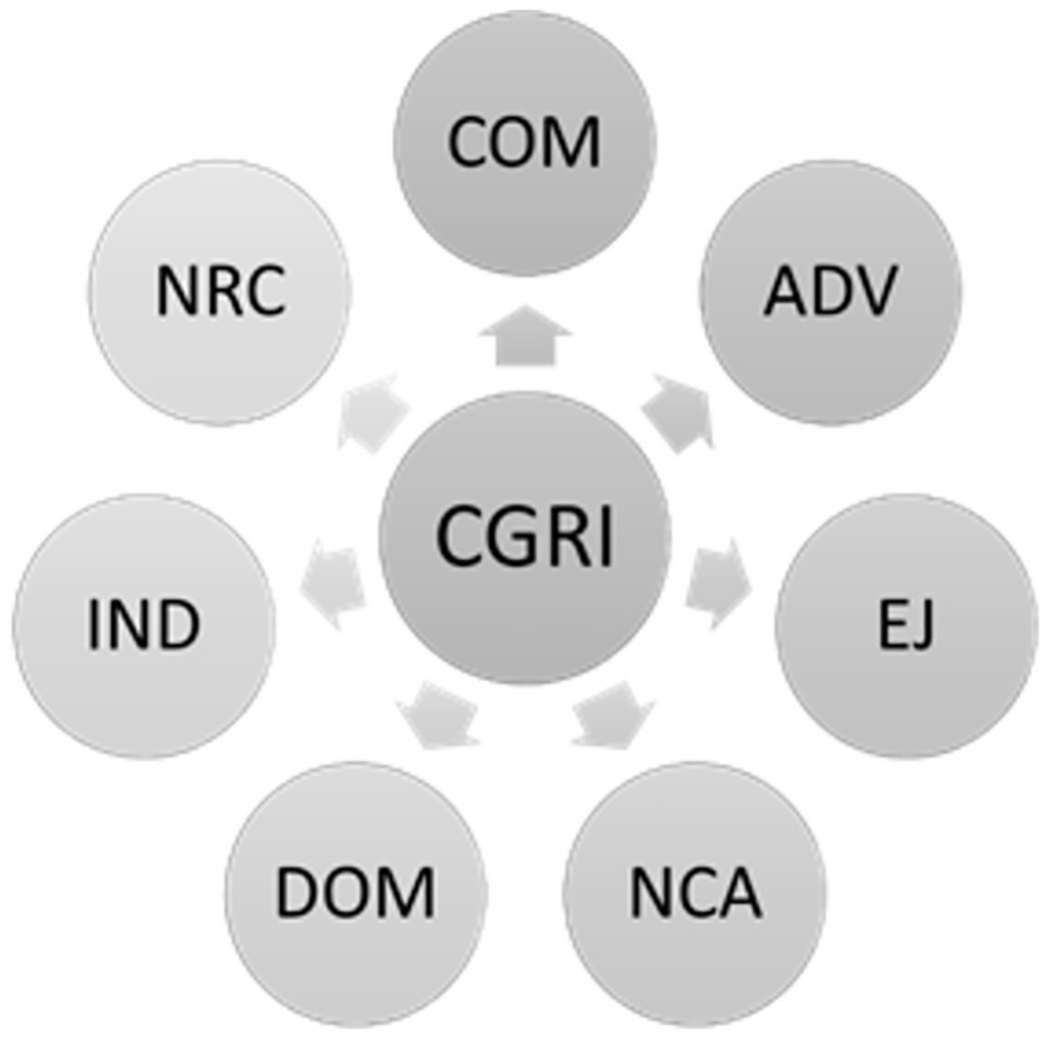
Measurement variables of the corporate CGRI.

A summary of the CGRI index variables can be found in Fig 1:

#### Control variables

Finally, based on [7], three control variables are included:

1. ***CNMV (Comisión Nacional del Mercado de Valores/National Stock Market Commision) sector*, *SECTOR (V***_***8***_). This variable controls which sectors obtain the best calculated CG results. In this case, it will be interesting to analyse what happens with companies that provide services, given their potential impact on other sectors. Each sector has its own characteristics, and it is desirable to study whether the effect of certain variables is greater in some sectors than in others. It is for this reason that the CNMV’s sector classification is used (Table 3).
2. ***Company size*, *SIZE (V***_***9***_***)*.** Larger companies are managed differently and therefore this control variable is included [22]. This variable is often used in governance studies and is defined as the logarithm of total assets. Larger companies are more frequently analysed than smaller companies, which may influence how they conduct their governance [22].
3. ***IBEX35*, *IBEX (V***_***10***_***)***. Ibex35 is the representative index of the SCM. This stock market index includes the 35 companies with the largest market capitalization in Spain. It is a dichotomous variable that will be 0 or 1 depending on the index membership in each corresponding year.

Table 4 shows descriptive values for the indicated variables. It is interesting to mention the dispersion of both the independent members and the dispersion found among the governance structure variables.

**Table 4:** Descriptive statistics for the variables used in the analysis.

	CGRI	COM	NRC	IND	EJ	NCA	ADV	DOM	Size
Mean	0,88	14,49	10,65	42,26	15,46	0,21	29,76	29,15	21,41
Standard Dev.	0,07	2,06	4,15	17,70	10,60	0,13	24,69	21,03	2,46
Minimum	0,67	0,00	0,00	0,00	0,00	0,00	0,00	0,00	14,74
Maximum	0,98	17,95	28,00	100,00	42,86	0,57	94,13	75,00	28,05

### 3.3 Empirical model

The use of the statistical technique of multiple linear regression makes it possible to generate a linear model in which the value of the dependent variable *(CGRI)* is determined from a set of independent variables (*V*_*h*_)

$$CGRI=\alpha +\sum_{h=1}^{10}\beta_{h}∙V_{h}+\varepsilon$$

Where:

*α*:Constant value. It is the intercept of the regression model.

*β*_*h*_:Linear regression coefficient. Represents the partial relationship of the *h-*th explanatory variable *h* = 1, 2, 3, …, 10 with the dependent variable. It is the average effect of a one-unit increase in the predictor variable *V*_*h*_ on the dependent variable *CGRI*, all other variables remain constant.

*ε*:Is the residual or error, i.e., the difference between an observed value and a value estimated by the model.

The aim is to identify all the explanatory variables *V*_*h*_ that explain the relationship and degree of association with the dependent variable *CGRI*, without any of them being a linear combination of the remaining variables. This can be achieved by using robust regression techniques (iteratively reweighted least squares) [98] where a weight is assigned to each observation, depending on whether they meet the assumptions underlying the standard multiple regression.

The application of this model is justified by its use in the literature either to predict a dependent variable’s value or to evaluate each predictor’s influence on it. In this way, the degree of influence of each variable in reference to the *CGRI* of SCM during 2018 and 2019 will be analysed.

## 4 Results by sector

The present research obtains a proposed model based on the stepwise regression procedure and the goodness of fit of the data to the multiple linear regression model. This model has the highest multiple correlation coefficient (*R*).

It is important to bear in mind that the magnitude of each partial regression coefficient depends on the units in which the predictor variable to which it corresponds (*V*_*h*_) is measured, so a coefficient’s magnitude is not associated with the importance of each predictor. In order to determine each variable’s impact on the model, standardised partial regression coefficients are used, which are obtained by standardising (subtracting the mean and dividing by the standard deviation) the predictor variables after adjusting the model (Table 5). In addition, it is observed that the third model has a Durbin-Watson value of 1.89 which is quite close to 2. This suggests that there is no significant evidence of autocorrelation in the residuals of the regression model. In practical terms, the model errors are practically independent of each other, which is a good sign for the validity of the model.

**Table 5:** Summary of the model^d^.

Model	R	R^2^	R^2^ adjusted	Standard error	Durbin-Watson
1	,455^a^	,207	,203	,06265716317	
2	,569^b^	,324	,317	,05802074858	
3	,587^c^	,344	,334	,05729879959	1,89

Statistical significance *p<*0.05

^a^. Predictors: (Constant), *SIZE*

^b^. Predictors: (Constant), *SIZE*, *DOM*

^c^. Predictors: (Constant), SIZE, DOM, NRC

^d^. Dependent variable: *CGRI*

From a general point of view, the variables that are related to higher governance indices are size, number of board meetings and number of CEOs. In the case of the first two variables, the relationship is directly proportional: the larger the company and the more meetings it holds, the higher the CGRI. In contrast, the greater the number of proprietary directors in a company, the lower the CGRI (Table 6).

**Table 6:** Coefficients^a^.

Model		Non-standard coefficients		Standard coefficients	*t*	Sig.
		*β*	Error	*β*		
1	(Constant)	,605	,040		15,016	,000
	*SIZE*	,013	,002	,455	6,995	,000
2	(Constant)	,664	,039		17,143	,000
	SIZE	,012	,002	,416	6,860	,000
	DOM	-,001	,000	-,344	-5,664	,000
3	(Constant)	,660	,038		17,222	,000
	SIZE	,011	,002	,379	6,126	,000
	DOM	-,001	,000	-,363	-5,999	,000
	NRC	,003	,001	,148	2,391	,018

Statistical significance *p<*0.05

^a^. Dependent variable: *CGRI*

Likewise, when taking into account the correlations between the variables defined in the present research and the CGRI, it can be observed that larger companies with higher director remuneration, with a greater number of annual meetings, with a higher percentage of independent governance directors and with a higher gender representation, obtain higher CGRIs. However, the number of proprietary directors has an inversely proportional influence on the index analysed (Table 7).

**Table 7:** Pearson correlation between the variables used in the analysis.

	CGRI	COM	NRC	IND	EJ	NCA	ADV	DOM	SIZE
CGRI	1,000								
COM	,244**	1,000							
NRC	,154*	0,137	1,000						
IND	,273**	0,127	,400**	1,000					
EJ	0,090	-,152*	-0,087	,249**	1,000				
NCA	,158*	0,089	0,132	,315**	0,102	1,000			
ADV	0,046	0,002	,301**	0,021	-0,016	0,092	1,000		
DOM	-,320**	0,016	,154*	-,352**	-,378**	0,021	,451**	1,000	
SIZE	,462**	,416**	,270**	,309**	-0,070	,279**	,205**	-0,068	1,000

Statistical significance

*p<0.05;

** *p<*0.01;

*** *p<*0.001

After the analysis of the 101 companies, the regression model was recalculated using the step-wise method, taking into account the productive sectors as a selection variable. It can be seen that in this case the R^2^ are higher. In this case, it is observed that, when analysing the data categorised on the basis of the productive sector, the variables influencing the CGRI differ mostly from the general regression model.

In the sectors of oil and energy (1); consumer goods (3); technology and telecommunications (6) and real estate services (7) it is shown that in order to achieve a good CGRI it is necessary to act on the variables that affect monitoring.

In the case of companies related to basic materials, industry and construction (2), it can be seen how the CGRI is influenced by variables that affect governance structure. On the other hand, the consumer services (4), technology and telecommunications (6) and real estate services (7) sectors relate their CGRI to the remuneration given to directors and executives. It should be mentioned that control variables have an effect, size is determinant in the oil and energy (1) and technology and telecommunications (6) sectors. In contrast, IBEX membership will be relevant in sectors basic materials, industry and construction (2), consumer goods (3) and consumer services (4) (Table 8).

**Table 8:** Summary of variable models by sector.

	Sector 1	Sector 2	Sector 3	Sector 4	Sector 5*	Sector 6	Sector 7
Constant	0,63	0,921	0,841	0,429		1,411	0,237
COM				0,035		0,022	0,035
NRC							0,014
IND			0,001			-,050	
EJ		-,003					
NCA	0,148						
ADV		0,002					
DOM		-,002	-,001	-,001			
SIZE	0,011					-,026	
IBEX		0,035	0,053	-0,520			
R	0,831	0,694	0,668	0,879		0,948	0,790
R^2^	0,690	0,481	0,446	0,772		0,898	0,624
R^2^ adjusted	0,643	0,434	0,397	0,743		0,859	0,585

Statistical significance *p<*0.05

## 5 Discussion

In contrast to what has been analysed to date in the literature related to CG, the data for companies listed on the SCM differ to a large extent from the results obtained in other studies and countries.

The main result obtained from the present research is when the sectoral breakdown is carried out, the data are very heterogeneous, which is the great contribution of this paper. Size has a positive effect on CG. The larger a company is, as is the case in the oil and energy sector, the better the mechanisms to provide an optimal degree of governance.

Although the literature argues that the remuneration of directors and top management may go against the interests of other stakeholders, it does not do so on the basis of CG, at least in large listed consumer goods companies. Maintaining good remuneration favours a higher CGRI index, particularly in the consumer services, technology and telecommunications and real estate services sectors of the SCM.

Previous studies on blockholders are very mixed in providing a positive or negative relationship on CG. They can have a positive effect on CG through direct intervention in the entity’s operations. However, they can have a negative effect if they acquire personal advantages, misappropriation or if their objectives are different from maximising the organisation’s value. Nevertheless, the results show that, for basic materials, industry and construction companies (around 25% of the companies analysed) the relationship of blockholders negatively affects CG.

In terms of executive members, several studies indicate that companies with smaller boards experience better governance. Thus, in consumer goods companies this is demonstrated by the fact that the larger the size, the lower the CG. On the other hand, in the case of technology and telecommunications companies, this effect is practically nil. Contrary to what has been argued in the literature so far, a higher number of proprietary directors does not positively affect CG, at least in oil and energy companies.

It is worth mentioning that most studies find a positive effect of independent directors on organisational performance, as they promote the choice of practices that, through monitoring, are intended to curb the possible systemic appropriation of blockholders and, therefore, favour the good development of CG. This is particularly true in companies related to consumer goods.

Surprisingly, and contrary to what the literature argues, the number of executive board members [94] and the incorporation of women into top management [99] do not affect the governance of these entities. In the first case, the disparity in SCM with high market capitalisation companies that have nothing to do with companies with returns below €500 million per year and with disparate governance structures could explain this situation. In the second case, information on the number of women in decision-making bodies is not included in a recommendation on a case-by-case basis. Therefore, when the companies analysed report information on this figure, even though they do not meet the minimum percentage of women required, this recommendation is considered to be covered. Recommendation 14 [37], apart from promoting the incorporation of women, also considers the approval of selection policies by the Board of Directors. It is suggested that this information should be broken down into two recommendations, in order to be able to reliably measure compliance with the incorporation of women in decision-making positions.

## 6 Conclusions

CG is a combination of policies, laws and instruments that influence the way a company is managed and conducted [1]. Until now, studies prior to this paper have focused on analysing the variables that affect CG through private indices. For the first time, an exhaustive analysis of which variables, defined throughout the literature, have a significant influence or not on good corporate governance is carried out through the CGRI which is derived from a public body such as the CNMV in Spain.

Likewise, there is a need to analyse CG on a sectoral basis, since the elements affecting CG vary greatly depending on the market in which they operate.

Finally, it should be noted that the CGRI prepared by the CNMV provides very high figures for SCM companies. Most of the companies analysed offer rates very close to 90% compliance. This may be due to the good CG of Spanish companies. Given that Spanish listed companies are obliged to report in an ACGR the degree of compliance with the recommendations and, if applicable, the explanation for the lack of compliance. The sufficiency and rigour of the explanations provided to justify non-compliance with some of these recommendations should be audited by external and independent professionals, so that shareholders, investors and the markets in general can properly judge them.

This research will be continued, as there is no doubt that there are links in common between Corporate Social Responsibility (CSR) and CG [100] and will have to be incorporated into the proposed index. In fact, [101] after conducting a literature review, evidence that there is a strong relationship between SCR and CG and come to propose a conceptual model that integrates the expectations of shareholders at the strategic level. In this way, both internal characteristics of the firm and the environmental context (institutions, regulation, dominant norms and values) are taken into account. As soon as there is mandatory reporting of this information, it can be included in the proposed index.
